# Comparative Label-Free Quantitative Proteomics Analysis Reveals the Essential Roles of N-Glycans in Salt Tolerance by Modulating Protein Abundance in *Arabidopsis*

**DOI:** 10.3389/fpls.2021.646425

**Published:** 2021-07-02

**Authors:** Chuanfa Liu, Guanting Niu, Xiaowen Li, Huchen Zhang, Huawei Chen, Dongxia Hou, Ping Lan, Zhi Hong

**Affiliations:** ^1^State Key Laboratory of Pharmaceutical Biotechnology, NJU Advanced Institute for Life Sciences (NAILS), School of Life Sciences, Nanjing University, Nanjing, China; ^2^Institute for Advanced Studies, Wuhan University, Wuhan, China; ^3^Research Center for Proteome Analysis, Shanghai Institutes for Biological Sciences, Chinese Academy of Sciences, Shanghai, China; ^4^State Key Laboratory of Soil and Sustainable Agriculture, Institute of Soil Science, Chinese Academy of Sciences (CAS), Nanjing, China

**Keywords:** N-glycan, salt response, label-free mass spectrum, proteomics, *Arabidopsis*

## Abstract

Many pieces of evidence show that the adaptive response of plants to salt stress requires the maturation of N-glycan on associated proteins. However, it is still little known about the salt-responsive glycoproteins that function in this process. In the present study, we identified salt-responsive glycoproteins in wild-type (WT) *Arabidopsis* and two mutants defective in N-glycan maturation, *mns1 mns2* and *cgl1*. A total of 97 proteins with abundance changes of >1.5‐ or <0.67-fold were identified against salt stress by label-free liquid chromatography coupled mass spectrometry (LC-MS/MS) quantitative analyses. A comparison of differentially abundant glycoproteins (DAGs) indicated the substrate preferences regulated by MNS1/MNS2 and CGL1. In addition, the DAGs in *mns1 mns2* hardly form functional regulatory networks in STRING analysis. Comparably, the regulatory network in *cgl1* was visible and shared overlapping with that in WT. Such difference may supply the evidence to partially explain the lower salt sensitivity of mutant *cgl1* than *mns1 mns2*. We further confirmed that two N-glycosylation clients, peroxidases PRX32 and PRX34, were involved in the salt stress response since the double mutants showed enhanced salt sensitivity. Together, our study provided proteomic evidence that N-glycans are crucial for modulating stress-responsive protein levels, and several novel glycoproteins responsible for salt stress tolerance in *Arabidopsis* were listed. Data are available *via* ProteomeXchange with identifier PXD006893.

## Introduction

As sessile organisms, land plants often suffer from various stresses and adverse environments, such as extreme temperatures, drought, and high salinity. In agriculture, high salinity affects the geographical distribution of crops in nature and causes a large amount of yield loss (Qin et al., 2011; Zhu, 2016). Extensive studies have revealed that a variety of genes and/or pathways play synergistic roles in abiotic stress response in plants, and it is difficult to significantly improve plant resistance to external stress by modulating a single effective gene or protein.

Protein asparagine (Asn or N)-linked glycosylation is one of the most conserved co‐ and post-translational modification (PTM) in eukaryotic cells (Khoury et al., 2011; Moremen et al., 2012). After the tetradecyl glycan precursor, GlcNAc_2_Man_9_Glc_3_ (Glc for glucose, Man for mannose, and GlcNAc for N-acetylglucosamine) is transferred to selected Asn residue in the Asn-X-Ser/Thr motif (X can be any amino acid residue except Pro) on nascent polypeptide chains (Pless and Lennarz, 1977; Yan and Lennarz, 2002) by oligosaccharyltransferase complex (OST; Kelleher and Gilmore, 2006), the N-linked oligomannosidic glycans are further trimmed and modified in Golgi apparatus to synthesize mature N-glycans with the secretion of associated polypeptides or proteins (Lerouge et al., 1998; Nagashima et al., 2018). In *Arabidopsis*, the initial N-glycan processing events in the Golgi are catalyzed by two functionally redundant class I α-mannosidases (MNS1 and MNS2), which cleave three α-1,2-mannosyl residues to generate the substrate for CGL1/GnT1 (Liebminger et al., 2009; Kajiura et al., 2010; Supplementary Figure S1A). The GnT1/CGL1 catalyzes a GlcNAc addition required to remove two additional Man residues to add another GlcNAc, xylose, and fucose residues to form a complex N-glycan structure (Vonschaewen et al., 1993; Strasser et al., 1999).

In plants, N-glycosylation is involved in many biological processes, including ER-quality control of steroid hormone receptor (Jin et al., 2007; Hong et al., 2008), gametophyte recognition (Lindner et al., 2015; Muller et al., 2016), subcellular transport (Rips et al., 2014; Shen et al., 2014), plant innate immunity (Li et al., 2009; Nekrasov et al., 2009; Haweker et al., 2010; Xia et al., 2020), and stomatal development (Jiao et al., 2020) by regulating the stability, function, or sub-localization of the substrate protein. Moreover, several studies also establish the association of N-glycosylation with salt tolerance in *Arabidopsis* (Koiwa et al., 2003; Kang et al., 2008; Nagashima et al., 2018). Of note, the failure of complex N-glycan biosynthesis leads to the salt sensitivity of *Arabidopsis* seedlings (Frank et al., 2008; Kang et al., 2008; Strasser, 2016; Liu et al., 2018). Intriguingly, when focusing on two *Arabidopsis* mutants, *mns1 mns2* (disrupted in Golgi MNSI) and *cgl1-3* (defected in GnTI/CGL1), in both of which glycoproteins harbor high mannose type N-glycans, we find that two mutants show a different degree of salt sensitivity (Liu et al., 2018). Although an increasing number of N-glycosylation-related mutants have been identified and show changes in response to salt stress (Strasser, 2016), yet it is still less known on the underlying molecular mechanism, mainly due to the lack of understanding of salt stress-responsive glycoproteins. Large-scale screening and identification of N-glycosylated sites and N-glycopeptides are now available (Zielinska et al., 2010; Stadlmann et al., 2017). A quantitative N-glycoproteomic pipeline has been established, and N-glycosylation signatures of different cell populations, including cancer cells, were recently reported for better stratification of cancer patients (Boyaval et al., 2020; Fang et al., 2020). N-glycoproteomes of insects are attracting scientists for pest control (Scheys et al., 2018). Inflorescence tissue-specific N-glycopeptides (Xu et al., 2016) and cold stress-regulated glycoproteins (Ma et al., 2016) are also resolved by mass spectrum (MS).

To explore the molecular mechanism of protein N-glycosylation regulating the salt stress response, we performed a label-free proteomic analysis utilizing nanoflow liquid chromatography coupled MS (nanoLC-MS/MS) and comparatively analyzed the proteome profiles between wild-type (WT) and two mutants against salt stress. We also confirmed that two differentially abundant peroxidases, PRX32 and PRX34, were N-glycosylation substrates and function redundantly in salt tolerance by root growth assay and hydrogen peroxide (H_2_O_2_) measurement.

## Materials and Methods

### Plant Materials and Growth Conditions

The *Arabidopsis thaliana* WT was the Col-0 ecotype. The mutant lines *mns1* (SALK_076002), *mns2* (SALK_023251), *prx32* (SALK_072340), *prx34* (SALK_051769), and *cgl1-3* (CS16367) mutant were purchased from ABRC (Arabidopsis Biological Resource Center at The Ohio State University). Double mutant *mns1 mns2* was described previously (Liu et al., 2018), and *prx32 prx34* was generated by cross *prx32* with *prx34* single mutant. Seed sterilization and plant growth conditions were described previously (Li et al., 2001).

### Salt Treatment

The *Arabidopsis* seeds were surface-sterilized and sown on half-strength Murashige and Skoog (MS) solid medium [2.2 g dm^−3^ MS salts (Duchefa, BH Haarlem, The Netherlands), 10 g dm^−3^ sucrose, and 3 g dm^−3^ Gelrite (Duchefa, BH Haarlem, The Netherlands), pH 5.7–5.9]. After cold treatment at 4°C for 2 days, the plates were placed in a 22°C growth chamber with 16-h light/8-h dark photoperiod for 12 days, and then cultured in 1/2 MS liquid medium or 1/2 MS medium containing 200 mM NaCl for 6 h, respectively. For MS analysis, the seedlings were collected and weighed to ensure that each sample was more than 2 g.

### Protein Extraction, Endo H Digestion, and Immunoblot Analysis

The plant materials including *Arabidopsis* seedlings and tobacco leaves were ground into fine powder in liquid N_2_ and added same volume of SDS loading buffer [125 mM Tris-HCl (pH 6.8), 4% (w/v) SDS, 20% (v/v) glycerol, 100 mM DTT, and 0.002% (w/v) bromophenol blue]. The extracts were thoroughly mixed by vortex and maintained on ice for 10 min. After heated at 95°C for 5 min, the mixture was centrifuged for 5 min at 16,000 *g*. The supernatant was treated with or without Endo Hf (New England Biolabs) treatment for 1.5 h at 37°C. Samples were then separated by 10 or 12% sodium dodecyl sulfate polyacrylamide gel electrophoresis (SDS-PAGE). The total proteins from the supernatants were separated by 10% SDS-PAGE. The immunoblot analyses were performed with primary antibodies, including anti-BRI1 (Mora-Garcia et al., 2004), anti-RSW2 (Liu et al., 2018), and HRP-conjugated goat anti-rabbit IgG secondary antibody (Promega). For PRX32-HA or PRX34-HA detection, immunoblot analyses were performed with peroxidase-conjugated anti-HA antibodies (Sigma). The protein signals were detected with enhanced chemiluminescence Immobilon Western HRP Substrate (Millipore) or SuperSignal West Pico PLUS substrate kit (Thermo) by a CCD imager (Tanon).

### Protein Preparation for MS Analysis

Total proteins were extracted with the modified trichloroethanoic acid (TCA) method described previously (Isaacson et al., 2006). Briefly, plant materials were ground into fine powder in liquid nitrogen and then lysed in 40 ml of cold TCA/acetone (v/v = 1/9, containing 65 mM DTT) solution, incubated at −20°C overnight. After centrifugation at 4°C at 10,000 *g* for 45 min, the supernatant was discarded, and the pellet was resuspended in 25 ml cold acetone. After centrifuged at 10,000 *g* for 45 min at 4°C again, the pellet was washed twice with cold acetone and dried in air. A total of 400 μl of UA buffer (8 M Urea, 150 mM Tris-HCl, pH 8.0) was used to dissolve the pellet before sonication. Finally, the supernatant was collected by centrifugation at 14,000 *g* for 30 min. Protein concentrations were determined by Bradford assay using bovine serum albumin as a standard (Bradford, 1976). The quality of the protein preparation was further evaluated by SDS-PAGE and visualized by Coomassie Brilliant Blue R-250 staining.

### Protein Digestion and LC-MS/MS Analysis

An aliquot of 400 μg of protein was digested according to the filter aided sample preparation (FASP) procedure (Zielinska et al., 2010). After adding 30 μl of SDT buffer (4% SDS, 100 mM Tris-HCl, 100 mM DTT, pH 7.6), the clear supernatants obtained from the above procedure were boiled for 10 min to 30 kDa ultrafiltration units (Wisniewski et al., 2011). Filters were rinsed three times with 200 μl of UA buffer (8 M Urea, 150 mM Tris-HCl, pH 8.0) and then incubated in UA buffer with the addition of 100 μl 50 mM iodoacetamide for 30 min in the dark. Subsequently, the filters were washed three times with 100 μl UA buffer and 100 μl 40 mM ammonium bicarbonate, respectively. Next, the proteins were digested with 8 μg trypsin at 37°C. After 16 h incubation, peptides were eluted and transferred to a new 30 kDa filtration unit. A total of 50 μl lectin mixture containing ConA (Sigma, United States), WGA (Sigma, United States), and RCA_120_ (Sigma, United States; 125 μg each) were added to the top of the filters. After incubation for 1 h, 25 mM NH_4_HCO_3_ in H_2_^18^O was used to wash unspecific peptides (Cambridge Isotope Laboratories, United States) twice. Deglycosylation with PNGase F (Roche, Switzerland) was performed in H_2_^18^O at 37°C for 3 h. Within H_2_^18^O, the deamidation conversion from Asn to Asp has a 2.9890 Da increase in molecular weight, which is the signature that distinguishes N-glycosylated peptides from O-glycosylated ones and those peptides that are not modified endogenously but survive the enrich anyway.

The deglycosylated peptides were desalted and isolated using nanoliter flow rate capillary high-performance liquid chromatography (HPLC) Easy-nLC. The following gradient was used for the mobile phases [A, 0.1% formic acid acetonitrile aqueous solution (2% acetonitrile); B, 0.1% formic acid acetonitrile aqueous solution (84% acetonitrile)]: 0–45% of B over 0–100 min; 45–100% of B over 100–108 min; and 100% of B over 108–120 min. Purified peptide mixtures were analyzed by mass spectrometry using a Q-Exactive mass spectrometer (Thermo Fisher Scientific, United States). Precursor MS scans were acquired with a resolution of 70,000 at m/z 200. Ten most intense ions were fragmented by higher-energy collisional dissociation (HCD) in the quadrupole collision cell. The HCD fragment ion spectra were acquired in the orbitrap with a resolution of 17,500 at m/z 200. The following conditions were used: automatic gain control (AGC) target: 3e6, maximum ion accumulation times of 20 ms for full scans, and 60 ms for HCD.

### Data Analysis

MS RAW data were searched against the TAIR10 protein database (Lamesch et al., 2012) in MaxQuant_1.3.0.5. The search followed an enzymatic cleavage rule of Trypsin/P and allowed a maximum of two missed cleavage sites and a peptide mass tolerance of 10 ppm. Carbamidomethylation of cysteine was defined as fixed modification, whereas protein N-terminal deamidation and methionine oxidation were defined as variable modifications for the database searches. The site false discovery rate (FDR) for peptide ≤0.01 (Zielinska et al., 2012). Label-free quantification was carried out using the MaxQuant software as described previously, and the intensity-based absolute quantification (iBAQ) of the identified peptides was selected to quantify protein abundance (Cox and Mann, 2008).

### Experimental Design and Statistical Rationale

For label-free quantitative analyses, all six biological samples were analyzed with three replicates. N-glycopeptides identified in two or more independent analyses were accepted to quantify the abundance of the N-glycopeptide. The abundance changes of unique peptides were set to represent the corresponding glycoprotein profile in response to salt stress. In the case of more than one unique peptide were detected within a glycoprotein, the abundance was presented as an average value.

### Bioinformatic Analysis

Candidate genes were classified with the online servers Agrigo (Du et al., 2010).^1^ Protein pathway analysis was performed using KEGG.^2^ The protein–protein interaction was analyzed with STRING.^3^

### Root Growth Assay

Experiment procedures were as described previously (Kang et al., 2008). Briefly, surface-sterilized seeds were sown on cellophane membrane and placed on MS agar medium (4.4 g dm^−3^ MS salts, 30 g dm^−3^ sucrose, and 16 g dm^−3^ agar, pH 5.7–5.9). After cold treatment at 4°C for 2 days, the plates were incubated at 22°C for 3 days, and then seedlings together with the cellophane membrane were transferred to MS medium or MS medium supplemented with 160 mM NaCl for another 9 days. The seedlings were photographed with a Canon G12 digital camera, and root lengths were measured by ImageJ software.^4^

### Reverse Transcription-PCR and Quantitative Real-Time PCR

Two-week-old WT, *prx32*, and *prx34* mutant seedlings were collected for genotyping by reverse transcription-PCR (RT-PCR). Salt-treated samples were harvested for *PRX32* and *PRX34* gene expression analysis by quantitative real-time PCR (qPCR). Plant materials were extracted by Trizol (Invitrogen, United States), 1 μg total RNA from each sample was used to synthesize cDNA by PrimeScript 1st Strand cDNA Synthesis kit (Takara, Japan). qPCR was performed in triplicate using the LightCycler® 96 System (Roche Life Science) with SYBR green mix (TaKaRa). Gene expression of *Actin2* was selected as an internal reference. The primers used for transcript amplification were listed as follows, ACT2-F (5'-GCCATCCAAGCTGTTCTCTC-3') and ACT2-R (5'-GCTCGTAGTCAACAGCAACAA-3') for *Actin2*, PRX32-F1 (5'-CTTCACTCTCCCACAACT-3') and PRX32-R1 (5'-TCGACCACATCATGTAGCA-3') for *PRX32*, and PRX34-F2 (5'-GTTGGTCTCGATCGTCCT-3') and PRX34-R2 (5'-ATGGAGCAGAGAGTTGGA-3') for *PRX34*.

### Transient Expression of PRX32 and PRX34 Proteins and Tunicamycin Treatment

To generate PRX32 and PRX34 protein expression cassettes, the full-length coding sequences of *PRX32* and *PRX34* with 1× HA tag at C-terminus were obtained by PCR using cDNA from WT plant as a template with following primers, PRX32-F (5'-TCCCCCGGGCTGCAGGAATTCATGAATTTCTCTTATTCTTCCTTGT-3') and PRX32-R (5'-GATAAGCTTGATATCGAATTCAGCGTAATCTGGAACATCGTATGGGTACATAGAGCTGACAAAGTCAACGA-3'), PRX34-F (5'-TCCCCCGGGCTGCAGGAATTCATGCATTTCTCTTCGTCTTCAACA-3') and PRX34-R(5'-GATAAGCTTGATATCGAATTCAGCGTAATCTGGAACATCGTATGGGTACATAGAGCTAACAAAGTCAACGA-3'). The PCR products were cloned into binary vector pQG110 *via* Gibson Assembly (NEB). The resulted constructs were transformed to *Agrobacterium tumefaciens* GV3101. The bacteria were cultured, collected, and suspended in IFB buffer [0.5% (w/v) Glucose, 10 mM MgCl_2_, 10 mM MES, and 150 mM acetosyringone, pH 5.7] at 0.5 OD_600_. The bacterial mixture was then infiltrated into tobacco *Nicotiana benthamiana* leaves using a syringe. The injected plants were cultured for 30–48 h before harvesting for protein extraction. For tunicamycin treatment, 5 μM tunicamycin (Sigma) solution was injected into the same infiltration areas 12 h before harvest.

### H_2_O_2_ Detection by DAB Staining

3,3'-Diaminobenzidine (DAB) staining was conducted as described previously (Daudi and O’Brien, 2012) with modifications. In brief, 2-week-old seedlings were cultured in 1/2 MS liquid medium containing 0 or 200 mM NaCl for 6 h, respectively. Seedlings were then washed with ddH_2_O three times before adding 5 ml of fresh 0.1% (w/v) DAB in 10 mM Na_2_HPO_4_ solution. After gently vacuum for 5 min to infiltrate the leaves, samples were incubated on a laboratory shaker for 4–5 h at 80–100 rpm in the dark. The chlorophyll was carefully bleached by boiling for 15 min before recording the staining results. H_2_O_2_ contents were quantified by grayscale analysis in ImageJ software.

## Results

### Identification of Glycosylated Peptides and Proteins Using LC-MS/MS

**Figure 1:**
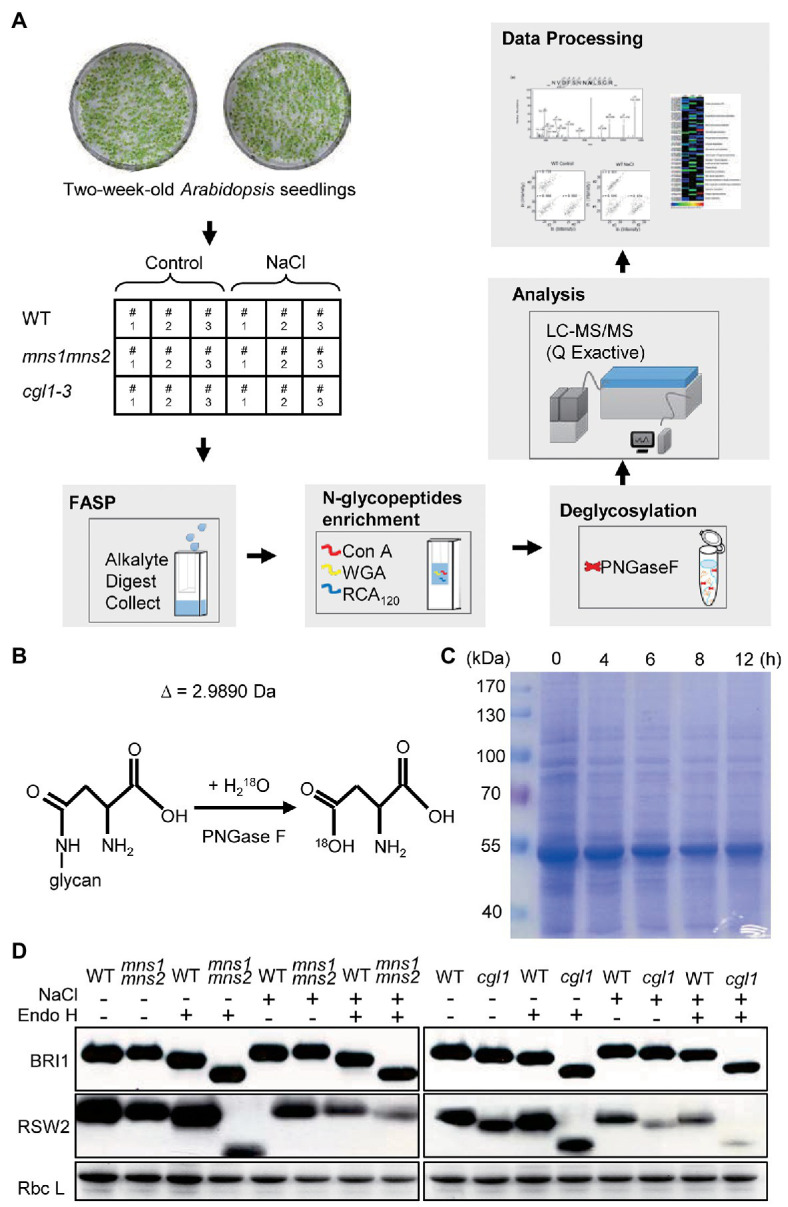
Schematic workflow of the experimental procedure. **(A)** A summary of the experimental design. Two-week-old seedlings were treated without (control) or 200 mM NaCl treatment (NaCl) for 6 h. Then, the total protein was extracted and digested into peptides using the filter aided sample preparation (FASP) method. N-glycopeptides were enriched by multiple mixed lectins and resolved by liquid chromatography coupled mass spectrometry (LC-MS/MS) after PNGase F digestion. The raw MS files were processed by MaxQuant. Three biological repeats were conducted. **(B)** Illustration of de-glycosylation of N-glycopeptides in H_2_^18^O by PNGase F. In the presence of H_2_^18^O, the conversion from Asn to Asp had a 2.9890 Da increase in molecular weight. **(C)** Detection of total protein abundance after salt treatment for different lengths of time. Two-week-old wild-type (WT) seedlings were treated in 200 mM NaCl for 0, 4, 6, 8, or 12 h. Total proteins were extracted and separated by 10% sodium dodecyl sulfate polyacrylamide gel electrophoresis. Gels were stained by Coomassie Brilliant Blue R-250 (CBB). **(D)** N-glycan structure analyses by Endo H digestion. Total proteins were extracted from control and salt-treated seedlings. After Endo H digestion, the proteins were separated on 10% SDS-PAGE and used anti-BRI1 and anti-RSW2 antibodies to analyze protein-associated N-glycan. CBB staining was shown as a loading control.

Two mutants *mns1 mns2* and *cgl1-3*, together with the WT, were employed for comparative analyses. In both *mns1 mns2* and *cgl1-3* mutants, complex N-glycan modification was blocked. Therefore, glycoproteins were decorated with oligomannosidic N-glycans. This feature facilitates the enrichment of glycopeptides by lectins more efficiently (Zielinska et al., 2010; Song et al., 2011). The workflow for label-free quantitative N-glycoproteomic analysis and the principle for N-glycosites identification was shown (Figures 1A,B). The NaCl concentration and treatment duration were optimized by phenotype observation and protein abundance measurements (Figure 1C). Eventually, 200 mM NaCl for 6 h was used as the salt treatment condition. We also detected the band shifts of two glycoproteins BRI1 and RSW2 against Endo H treatment to assess if the salt treatment would block the N-glycan structure under such conditions. The results showed that the digested band patterns of two proteins did not change either in the WT or in the mutant background before and after salt treatment (Figure 1D), indicating that salt treatment had no significant effect on the structure of N-glycan, thus excluding the possible effect of N-glycan structural changes on protein enrichment in our study. After proteins were extracted, denatured, and digested with trypsin, the N-glycopeptides were enriched using FASP-based multiple lectins approach (Zielinska et al., 2010). To capture all three classes of high-mannose, hybrid, and complex types of N-glycosylated peptides (Supplementary Figure S1A), multi-lectin enrichment was employed. Multiple lectins were composed of concanavalin A (ConA), wheat germ agglutinin (WGA), and castor lectin RCA_120_, which bound mannose, N-acetylglucosamine, and galactose residues, respectively, on N-glycans (Supplementary Figure S1B). We first examined LC-MS/MS performance and the quality of the dataset. Pearson correlation analysis showed that the intensities of N-glycopeptides in three replicates for each *Arabidopsis* line, either with or without salt treatment, were highly correlated (*r* = 0.646–0.931; Supplementary Figure S2). We also assessed the number of identified N-glycopeptides and N-glycoproteins and found that more than half of N-glycopeptides were identified from three (40.45–57.27%) or two (57.30–80.77%) independent experiments (Supplementary Figure S3). These results showed that the N-glycoproteomic data were suitable for further analysis.

**Figure 2:**
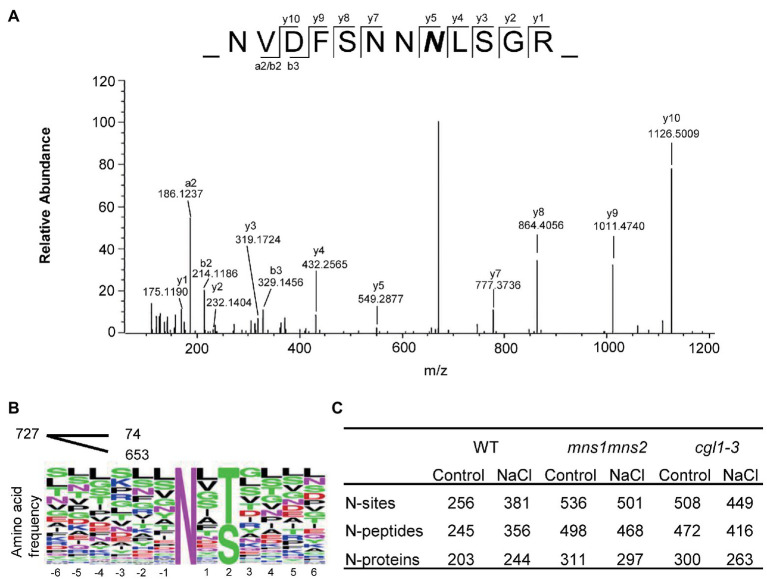
An overview of identified N-glycosylated motifs, peptides, and proteins. **(A)** Representative MS/MS spectrum of the peptide NVDFSNNNLSGR of the EF-Tu receptor (EFR). The 2.9890 Da mass increment of deamidation is indicated by a bold italic *N*. **(B)** Consensus sequence of identified N-glycosylation sites. Multiple glycosylated sequence motifs were analyzed, and relative frequency plots are shown. Of the total 727 sequence motifs, 74 are noncanonical motifs with an occurrence of <20 each. **(C)** Statistics of identified N-glycosites, N-glycopeptides, and N-glycoproteins by three replicates in six sample groups.

A total of 727 glycosylation sites from 632 glycosylated polypeptides were identified, corresponding to 371 glycoproteins. Most N-glycosylation sites exhibited canonical N-X-S/T motif sequences (Figures 2A,B). The number of identified N-glycoproteins varied among samples. In control groups, 311 glycoproteins were identified in *mns1 mns2*, and 300 glycoproteins were identified in the *cgl1-3* mutant, while only 203 were identified in the WT (Figure 2C). Different glycoproteins would be enriched despite the use of saturated multiple lectins, e.g., ConA bound more proteins from *mns1 mns2* and *cgl1* than the WT. Similarly, PNGase F was less effective on N-glycans from the WT. Therefore, in the WT, the reduced N-glycoproteins might be because the glycoproteins carrying complex N-glycans were less enriched than those with high mannose type N-glycans in the mutants. However, in the presence of high salinity, the number of glycoproteins detected in the WT slightly increased (from 203 to 244) but decreased in both *mns1 mns2* (from 311 to 297) and *cgl1-3* (from 300 to 263; Figure 2C). One possibility is that the abundance of glycoproteins in response to salt stress was modulated by the attached N-glycan structure and maturation failure of N-glycans destabilized the associated glycoprotein under salt stress, which corresponded to previous studies in which the maturation of N-glycans was essential for plants to tolerate salt stress.

### Characterization of Identified Glycoproteomes by GO Annotation

The function of identified N-glycoproteins was analyzed by gene ontology (GO) annotation from the following aspects: the participation of biological processes (BPs), molecular function (MF), and cell components (CCs; Ashburner et al., 2000). In general, the identified N-glycoproteins were involved in various BPs, including protein and carbohydrate metabolic processes, anatomical structure morphogenesis, photosynthesis, cellular development process, regulation of cell size, and stress response. For CC, the subcellular locations of identified proteins were in line with the expectation; the proteins were mainly secretory or membrane proteins and were distributed in the extracellular and endomembrane, including the endoplasmic reticulum (ER), Golgi apparatus, and vacuole, most of which undergo N-glycosylation. MF analysis showed that the identified N-glycosylated proteins were concentrated in catalytic, hydrolase, transferase, and kinase activities (Supplementary Figure S4). We further compared the glycoproteome profiles of the WT, *mns1 mns2*, and *cgl1-3* between the salt treatment and corresponding control group. The results showed no significant difference in enriched GO items under salt stress (Supplementary Table S1). However, we found that more glycoproteins were detected in *mns1 mns2* and *cgl1-3* mutants than in the WT, which were mainly enriched in kinase and transferase activities (Supplementary Table S1), inferring that some kinases and transferases are significantly affected by the associated N-glycan structures under salt stress.

### Global Changes of Protein Abundance in Response to Salt Stress

To further evaluate the effects of N-glycans on salt stress-responsive glycoproteins, we quantitatively compared the alterations of the N-glycoproteome. We set a threshold of protein abundance change to 1.5-fold, meaning that a protein with an abundance ≤0.67‐ or ≥1.5-fold after salt treatment was regarded as a salt responsive protein. In the WT, 37 of 141 proteins were quantified as salt responsive, of which 32 proteins increased by more than 1.5-fold and five proteins decreased to less than 0.67-fold. In mutants, the abundance of 45 of 236 glycoproteins in *mns1 mns2* changed, of which 33 proteins accumulated and 12 proteins were decreased. Similarly, the abundance of 42 of 207 glycoproteins was changed in *cgl1-3*, of which 13 members were increased, and most members were decreased (Table 1). These analyses showed that the abundance of most salt-responsive proteins was increased in the WT compared to the mutants, particularly in *cgl1-3*, in which most glycoproteins were decreased. We also made a qualitative comparison between control and NaCl-treated samples. We defined a peptide/protein with quantitative values as presence, and otherwise, it was regarded as an absence. Pairwise analyses showed that three proteins (Glycosyl hydrolase family protein, BGLU34, EBP1) were exclusively detected in the WT control group, while eight proteins of the 11 exhibiting catalytic activity were only detected in the salt-treated WT. Similarly, eight proteins were only detected in the *mns1 mns2* control group, and nine unique proteins, including BGLU24, FUC1, LYM1, IAR3, VSP2, sks3, PAP17, and two unknown proteins, were detected in salt-treated *mns1 mns2*; meanwhile, 21 proteins only presented in the *cgl1-3* control group and, most of which were involved in the carbohydrate metabolic process (Supplementary Table S2). Eight proteins were affected in the same way in both mutants: the abundance of HBP1, GLP10, FLA9, and PRX34 increased, while STT3A, XXT5, AT1G59970, and RHS19 decreased in both *cgl1-3* and *mns1 mns2*. In addition, three proteins, BGLU44, SCPL46, and PAP2, increased in *mns1 mns2* but decreased in *cgl1-3*. NAI2 was decreased in *mns1 mns2* but increased in *cgl1-3* (Table 2). In addition, there were more differentially abundant glycoproteins (DAGs) exclusively detected in *mns1 mns2* or *cgl1-3* (Table 2). In conclusion, although the variation trends of DAGs in *mns1 mns2* and *cgl1-3* were similar, they were also different, which may provide clues to explain the different salt responses between the two mutants.

**Table 1:** Statistical analysis of differentially abundant N-glycopeptides and N-glycoproteins in response to salt stress.

NaCl: control	# of quantified		# of regulated (fold changes > 1.5, *p* < 0.05)			
	N-glyco-peptides	N-glyco-proteins	N-glycopeptides^*^		N-glycoproteins^*^	
WT	163	141	40 ↑	4 ↓	32 ↑	5 ↓
*mns1 mns2*	340	236	36 ↑	17 ↓	33 ↑	12 ↓
*cgl1-3*	294	207	12 ↑	51 ↓	13 ↑	29 ↓

∗ ↑ indicates NaCl: control ratio > 1.5; ↓ indicates NaCl: control ratio < 0.67.

**Table 2:** Differentially abundant glycoproteins were identified in the WT, *mns1 mns2*, and *cgl1-3* in response to salt stress.

Accession number	Protein descriptions	Ratio^*^ (T/NT)	Ratio^*^ (D/ND)	Ratio^*^ (C/NC)
**Post-translational modification, protein folding, and chaperones (10)**				
AT1G56340	Calreticulin 1a, CRT1a	2.14 ± 0.19	-	-
AT1G09210	Calreticulin 1b, CRT1b	0.13 ± 0.02	-	-
AT2G38960	Endoplasmic reticulum oxidoreductins 2, ERO2	2.17 ± 0.03	-	-
AT3G07770	Heat shock protein 89.1, Hsp89.1	-	3.60 ± 1.30	-
AT3G54960	PDIL1-3, a protein disulfide isomerase-like protein	1.91 ± 0.28	-	-
AT4G27080	PDI-like 5-4, PDI7, PDIL5-4	-	-	0.24 ± 0.03
AT5G19690	Staurosporin and temperature sensitive 3-like A, STT3A	2.03 ± 0.22	0.56 ± 0.05	0.26 ± 0.05
AT1G76400	Ribophorin I, an oligosaccharyl transferase subunit	2.23 ± 0.47	-	-
AT1G67490	Glucosidase 1, GCS1, KNF	-	2.54 ± 0.28	-
AT1G71220	UDP-glucose:glycoprotein glucosyltransferases, EBS1	2.78 ± 0.71	-	0.41 ± 0.18
**Carbohydrate metabolism and energy production (16)**				
AT3G26650	GAPDH A subunit, GAPA	0.29 ± 0.16	-	0.25 ± 0.02
AT1G42970	GAPDH B subunit, GAPB	-	-	0.37 ± 0.17
AT1G09780	Phosphoglycerate mutase	0.32 ± 0.16	-	-
ATCG00680	Photosystem II reaction center protein B, PSBB	0.32 ± 0.10	-	0.08 ± 0.00
ATCG00480	ATP synthase subunit beta, ATPB	-	3.39 ± 0.38	-
AT2G28470	Beta-galactosidase 8, BGAL8	2.67 ± 0.13	-	0.34 ± 0.20
AT5G63810	Beta-galactosidase 10, BGAL10	1.85 ± 0.11	-	0.42 ± 0.13
AT5G13640	Phospholipid:diacylglycerol acyltransferase, PDAT	2.19 ± 0.30	-	-
AT1G66970	SVL2, a member of GDPD-like family	-	-	0.27 ± 0.17
AT1G74380	Xyloglucan xylosyltransferase 5, XXT5	-	0.52 ± 0.06	0.28 ± 0.02
AT3G26720	Glycosyl hydrolase family 38 protein	-	-	0.52 ± 0.12
AT2G44450	Beta glucosidase 15, BGLU15	-	0.23 ± 0.09	-
AT1G52400	Beta glucosidase 18, BGLU18	2.57 ± 0.37	2.21 ± 0.59	-
AT3G18080	B-S glucosidase 44, BGLU44	-	3.36 ± 0.36	0.25 ± 0.02
AT1G65590	Beta-hexosaminidase 3, HEXO3	-	-	4.63 ± 0.97
AT1G07230	Non-specific phospholipase C1, NPC1	-	4.31 ± 0.71	-
**Putative uncharacterized protein (14)**				
AT5G14030	TRAPB family protein	0.54 ± 0.11	0.49 ± 0.10	-
AT5G15350	Early nodulin-like protein 17, ENODL17	1.52 ± 0.02	-	-
AT4G12880	Early nodulin-like protein 19, ENODL19	-	1.55 ± 0.03	-
AT3G62730	Unknown protein, desiccation-like protein	3.99 ± 0.41	-	-
AT2G12400	Unknown protein, plasma membrane fusion protein	4.22 ± 0.60	-	-
AT1G19370	Unknown protein, membrane protein	-	0.07 ± 0.00	-
AT5G58100	Unknown protein, transmembrane protein	-	0.28 ± 0.05	-
AT3G56750	Unknown protein, transferase activity	-	2.38 ± 0.23	-
AT1G05070	Protein of unknown function (DUF1068)	-	1.76 ± 0.15	-
AT2G01310	Hypothetical protein	-	-	3.60 ± 0.32
AT3G13410	2-C-methyl-D-erythritol 4-phosphate cytidylyltransferase	-	2.23 ± 0.35	-
AT1G10950	Transmembrane nine 1, TMN1	-	-	0.21 ± 0.15
AT1G09870	Histidine acid phosphatase family protein	-	1.59 ± 0.26	-
AT1G17100	HBP1, SOUL heme-binding family protein	-	2.30 ± 0.03	1.59 ± 0.16
**Protein transport and metabolism (9)**				
AT2G35780	Serine carboxypeptidase-like 26, SCPL26	1.67 ± 0.37	-	-
AT5G23210	Serine carboxypeptidase-like 34, SCPL34	-	2.83 ± 0.96	-
AT2G33530	Serine carboxypeptidase-like 46, SCPL46	-	1.86 ± 0.23	0.62 ± 0.09
AT1G78680	Gamma-glutamyl hydrolase 2, GGH2	2.73 ± 0.71	-	-
AT3G62020	Germin-like protein 10, GLP10	3.74 ± 0.59	3.85 ± 1.27	3.56 ± 0.06
AT1G59970	Matrixin family protein	-	0.55 ± 0.00	0.60 ± 0.04
AT1G13900	PAP2, a dual-localized acid phosphatase that modulates protein targeting to mitochondrion	3.77 ± 0.01	2.27 ± 0.45	0.47 ± 0.16
AT3G52850	Vacuolar sorting receptor homolog 1, VSR1	-	3.22 ± 0.68	-
AT4G20110	Vacuolar sorting receptor 7, VSR7	-	2.55 ± 0.02	-
**Defense response (11)**				
AT1G79340	Metacaspase 4, MC4, MCP2d	2.02 ± 0.32	3.17 ± 0.40	-
AT1G52410	TSK-associating protein 1, TSA1	2.88 ± 0.63	-	-
AT2G39730	Rubisco activase, RCA	3.02 ± 0.05	-	-
AT5G06860	Polygalacturonase inhibiting protein 1, PGIP1	-	-	0.49 ± 0.04
AT5G06870	Polygalacturonase inhibiting protein 2, PGIP2	-	3.53 ± 0.70	-
AT3G11650	NDR1/HIN1-like 2, NHL2	-	-	1.59 ± 0.11
AT5G37780	CAM1, calmodulin 1	-	-	11.11 ± 2.63
AT5G26000	Thioglucoside glucohydrolase 1, TGG1, BGLU38	1.80 ± 0.17	-	-
AT5G25980	Glucoside glucohydrolase 2, TGG2, BGLU37	-	2.84 ± 0.34	-
AT3G14210	Epithiospecifier modifier 1, ESM1	2.13 ± 0.24	1.50 ± 0.17	-
AT1G08470	Strictosidine synthase-like 3, SSL3	-	-	0.43 ± 0.15
**Cell wall biosynthesis and modification (14)**				
AT5G49720	Endo-1,4-glucanase, GH9A1, KORRIGAN/RSW2	2.13 ± 0.15	-	0.28 ± 0.15
AT3G14310	Pectin methylesterase 3, PME3	-	-	2.45 ± 0.43
AT4G33220	Pectin methylesterase 44, PME44	-	2.32 ± 0.43	-
AT2G35610	Xyloglucanase 113, XEG113	-	0.27 ± 0.02	-
AT4G37800	Xyloglucan endotransglucosylase/hydrolase 7, XTH7	-	0.10 ± 0.03	-
AT3G16860	COBRA-like protein 8 precursor, COBL8	-	-	0.50 ± 0.11
AT1G68560	Alpha-xylosidase 1, XYL1	-	-	0.46 ± 0.03
AT5G64570	Beta-D-xylosidase 4, XYL4	-	-	0.49 ± 0.04
AT4G01080	TRICHOME BIREFRINGENCE-LIKE 26, TBL26	-	2.34 ± 0.35	-
AT5G55730	FASCICLIN-like arabinogalactan 1, FLA1	-	1.83 ± 0.10	-
AT2G04780	FASCICLIN-like arabinogalactan 7, FLA7	-	2.36 ± 0.36	-
AT1G03870	FASCICLIN-like arabinogalactan 9, FLA9	-	3.38 ± 0.17	1.97 ± 0.14
AT1G28290	Arabinogalactan protein 31, AGP31	-	2.90 ± 0.36	-
AT3G09090	Defective in exine formation protein, DEX1	-	-	0.46 ± 0.13
**Signal transduction (8)**				
AT1G28340	Receptor like protein 4, RLP4	2.35 ± 0.37	-	-
AT1G73080	PEP1 receptor 1, PEPR1, LRR receptor kinase	1.79 ± 0.29	-	-
AT3G51740	Inflorescence meristem receptor-like kinase 2, IMK2	3.34 ± 0.74	-	-
AT4G33430	BRI1-associated receptor kinase, BAK1	3.07 ± 0.87	-	0.54 ± 0.06
AT5G48380	BAK1-interacting receptor-like kinase 1, BIR1	-	-	0.48 ± 0.14
AT3G17840	Receptor-like kinase 902, RLK902	-	-	2.16 ± 0.03
AT1G21270	Wall-associated kinase 2, WAK2	-	0.64 ± 0.06	-
AT4G22130	STRUBBELIG-receptor family 8, SRF8	-	1.80 ± 0.28	-
**Oxidation-reduction process (11)**				
AT5G62630	hipl2 protein precursor, HIPL2	3.72 ± 0.70	-	-
AT5G63910	Farnesylcysteine lyase, FCLY	4.26 ± 0.81	3.11 ± 0.62	-
AT5G21105	Plant L-ascorbate oxidase	2.11 ± 0.28	-	1.95 ± 0.03
AT3G32980	Peroxidase 32, PRX32	-	-	2.04 ± 0.33
At3g49120	Peroxidase 34, PRX34	-	1.35 ± 0.05	1.34 ± 0.03
AT5G67400	Root hair specific 19, RHS19	-	0.27 ± 0.16	0.16 ± 0.03
AT1G74790	Catalytics	2.96 ± 0.18	-	-
AT2G01270	Quiescin-sulfhydryl oxidase 2, QSOX2	-	-	0.29 ± 0.04
AT1G76160	SKU5 similar 5, SKS5	-	-	0.31 ± 0.08
AT1G41830	SKU5-similar 6, SKS6	-	-	0.24 ± 0.03
AT4G39640	Gamma-glutamyl transpeptidase 1, GGT1	-	1.77 ± 0.27	-
**ER and ribosome biogenesis (2)**				
AT2G39780	Ribonuclease 2, RNS2, the main endoribonuclease activity in plant cells.	2.31 ± 0.33	-	-
AT3G15950	DNA topoisomerase related, NAI2, loss of function mutations lacks ER bodies.	-	0.54 ± 0.03	4.15 ± 0.27
**Auxin biosynthesis and signaling (3)**				
AT3G07390	Auxin-responsive family protein, AIR12	-	-	6.00 ± 0.39
AT4G24670	TAR2, auxin biosynthetic process	-	2.77 ± 0.28	-
AT4G02980	Auxin binding protein 1, ABP1	-	2.87 ± 0.72	-

∗ Indicates glycoprotein abundance relative to the control group upon NaCl stress in the WT (T/NT), *mns1 mns2* (D/ND), and *cgl1-3* (C/NC) background. ‐ denotes no significant abundance change or undetected. Notably, due to limited space, proteins with more than 1.5-fold changes are listed unless PRX34.

### Salt Responsive Glycoproteins Are Differentially Abundant in *mns1 mns2* and *cgl1* Mutants

**Figure 3:**
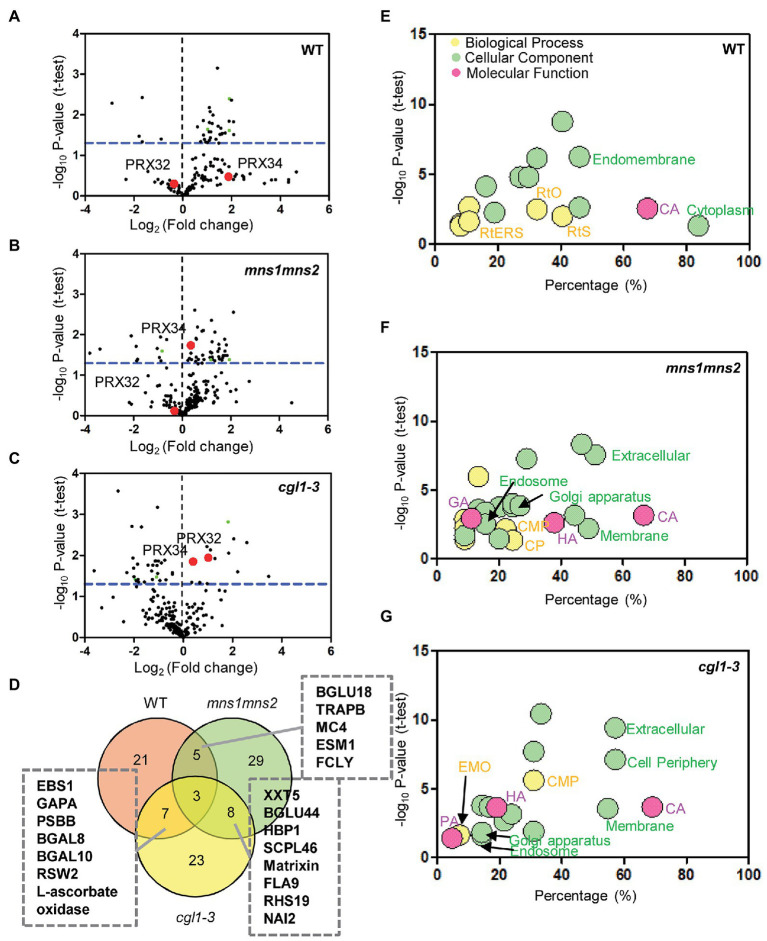
Gene ontology (GO) annotations of differentially abundant N-glycoproteins under salt stress. **(A–C)** Identification of differentially abundant N-glycoproteins in the WT **(A)**, *mns1 mns2* **(B)**, and *cgl1-3* **(C)** under salt stress. Each dot represents one protein. The blue horizontal bar represents the threshold value of *p* (≤0.05). The red dots represent PRX32 and PRX34. **(D)** Venn diagram demonstrating the overlap in the number of salt stress-responsive glycoproteins in the WT, *mns1 mns2*, and *cgl1-3*. Three common proteins quantified in the WT and two mutants are presented as green dots in **(A–C)**. **(E–G)** GO analyses of salt stress-responsive proteins in the WT **(E)**, *mns1 mns2* **(F)**, and *cgl1-3* **(G)**. Significantly enriched GO terms are marked on the diagram. CA, catalytic activity; HA, hydrolase activity; PA, phosphorylating activity; GA, glucosidase activity; RtO, response to organic substance; RtS, response to stress; RtERS, response to ER stress; CMP, carbohydrate metabolic process; CP, catabolic process; EMO, extracellular matrix organization.

The integral GO analysis of DAGs demonstrated that biological processes containing carbohydrate metabolic process, response to stress, and cellular component organization were modulated for salt tolerance (Supplementary Table S3). We further analyzed the biological functions of DAGs under salt stress in GO annotation and KEGG metabolic pathways. Consistent with the aforementioned results (Figure 2C), more decreased glycoproteins were observed in the two mutants, especially in *cgl1-3* (143 vs. 67 in *mns1 mns2*; Figures 3A–C; Table 1). Notably, hydrolase activity was enriched in both *mns1 mns2* and *cgl1-3* mutants compared with the WT (Figures 3D–F). Next, we aligned the DAGs from the WT, *mns1 mns2*, and *cgl1-3* with *p* < 0.05, and the abundance changes of the shared proteins were analyzed individually (Table 2). Under salt stress, the abundance of STT3A (OST Subunit), purple acid phosphatase 2 (PAP2), and germin-like protein 10 (GLP10) in the WT and two mutants changed, which are involved in the glycan chain transfer in the ER, root growth, and carbon metabolism, respectively (Figure 3D). STT3A increased in the WT but decreased in *mns1 mns2* and *cgl1-3*; PAP2 increased in the WT and *mns1mns2* but decreased in *cgl1-3*, and GLP10 increased in all three samples (Table 2). The commonly changed DAGs between the WT and *cgl1-3* were stress response proteins (EBS1, GAPA, and KORRIGAN/RSW2) and active catalytic proteins (EBS1, BGAL8, PSBB, GAPA, KORRIGAN/RSW2, and BGAL10). Comparably, those DAGs related to the glycosyl compound catabolic process (BGLU18, FCLY, and ESM1) were changed in both WT and *mns1 mns2*. The DAGs shared by *mns1 mns2* and *cgl1-3* were mostly located in the membrane and cell wall (XXT5, HBP1, FLA9, BGLU44, and SCPL46; Table 2; Figure 3D). The further molecular functional analysis found that hydrolase activity was enriched in *mns1 mns2* and *cgl1-3* compared with the WT (Figures 3E,G), and most of them participated in cell wall biosynthesis and modification (Table 2), indicating that cell wall integrity and/or composition are crucial for salt tolerance. In addition, the DAGs in mutants had a broader distribution, including the Golgi apparatus, endosome, ER, vacuole, cell membrane, and extracellular regions. In the WT, the DAGs were most prevalent in the cytoplasm and endomembrane systems. The biological process of proteins identified in the WT was significantly enriched in response to stress and organic substances, while most DAGs from the mutants were related to the carbohydrate metabolic process (Figure 3G). These results suggested that salt-responsive glycoproteins in *mns1 mns2* and *cgl1-3* mutants cannot respond to stress correctly, which may be due to the lack of correct N-glycan modification, resulting in their abnormal subcellular localization.

Two members from the class III peroxidase family identified in the DAGs, PRX32 (At3g32980) and Peroxidase 34 (PRX34, At3g49120). The protein abundance of neither PRX32 nor PRX34 was significantly changed in the WT under salt stress (Supplementary Table S5). However, both PRX32 and PRX34 were significantly increased in the *cgl1-1* mutant by more than 2-fold (*p* = 0.011429) and 1.3-fold (*p* = 0.014898), respectively. Interestingly, in the *mns1mns2* mutant, only PRX34, the more abundant protein, displayed a 1.3-fold increase (*p* = 0.018209; red point in Figures 3A–C; Supplementary Table S5). We reasoned that the absence of N-glycan modification may affect the functions of PRX proteins in the mutants, and the increase of protein abundance may be due to the feedback regulation under salt stress. While the different changes of the two proteins probably contribute to the difference in salt tolerance between *mns1 mns2* and *cgl1-3*.

KEGG pathway analysis of DAGs showed that proteins processing in the ER, biosynthesis of secondary metabolites, plant-pathogen interactions, N-glycan biosynthesis, and photosynthesis were influenced by salt stress in the WT and mutants (Supplementary Figure S5). However, *mns1 mns2* mutations mainly affected glycolysis/gluconeogenesis, biosynthesis of antibiotics, tryptophan metabolism, terpenoid backbone biosynthesis, and carotenoid biosynthesis. In contrast, in the *cgl1-3* mutant, plant hormone signal transduction, carbon metabolism, carbon fixation in photosynthetic organisms, ascorbate, aldarate metabolism, and microbial metabolism in diverse environments were predominantly enriched (Supplementary Table S4). Taken together, these results suggested that N-glycan modification mediated by MNS1/MNS2 and CGL1 may have different influences on salt-responsive glycoproteins to adapt to salt stress.

### Protein–Protein Interaction Network Analysis by STRING

**Figure 4:**
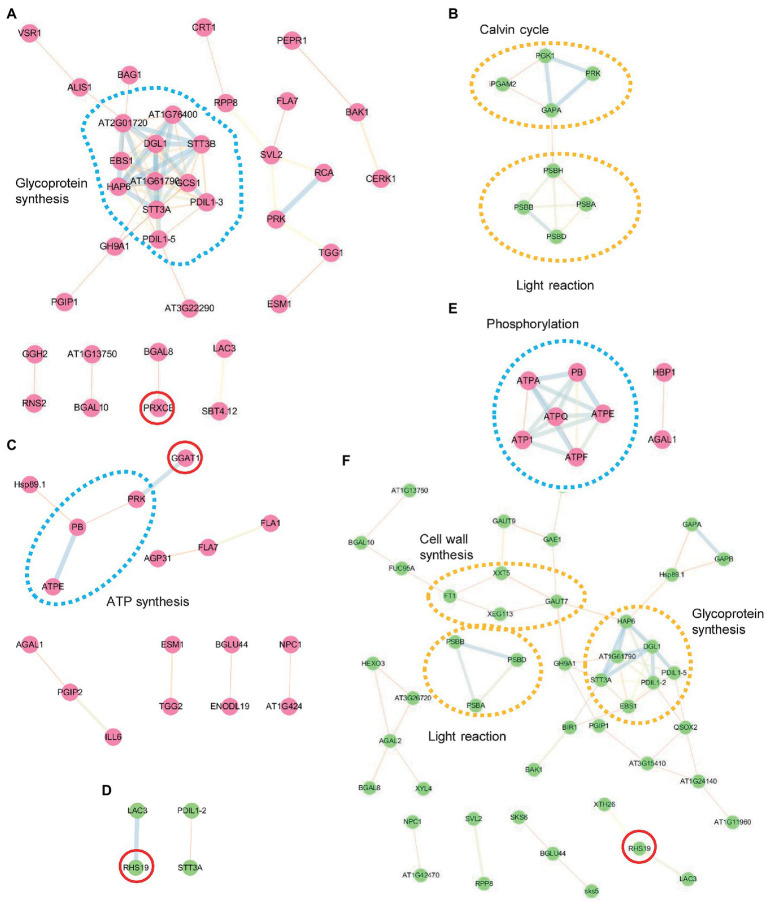
Protein interaction network analysis using the STRING database. Interaction network of differentially abundant glycoproteins (DAGs) was performed with the STRING system (https://string-db.org) based on known and predicted interactions. Protein–protein interactions among increased (red points) and decreased (green points) proteins in the WT **(A,B)**, *mns1 mns2* **(C,D)**, and *cgl1-3* **(E,F)** were analyzed separately. Lines of different font-weight represent different types of evidence for the associations. The dotted circles highlight the involved biological processes. Red circles are proteins associated with the oxidation-reduction process. Proteins without interactions are not shown.

N-glycoproteins include many secretory and membrane-bound proteins involved in the intracellular networks and intercellular communications of plant development and adaptive responses to stress. After identifying salt-responsive N-glycoproteins, we evaluated the possible regulatory network among them using the STRING server.^5^ We classified the identified proteins into two groups, increased and decreased under salt stress. In the WT, the increased proteins contained multiple molecular chaperones (PDIL1-3, PDIL1-3, and EBS1) and oligosaccharyltransferase subunits [STT3A, STT3B, At2g01720 (OST1A), At1g76400 (OST1B), HAP6, DGL1, and At1g61790 (OST3/6)] and present a close interaction for N-glycosylation, protein folding, and ER quality control (Figure 4A). In the decreased group, only several proteins related to the Calvin cycle and light reaction were illustrated (Figure 4B). In comparison, the DAGs in *mns1 mns2* hardly formed functional regulatory networks. This might be caused by decreased protein stability or abundance and made the detected proteins more dispersed (Figures 4C,D). In the *cgl1* mutant, the networks were still visible in the phosphorylation pathway by increased proteins (Figure 4E) and N-glycosylation biogenesis, cell wall organization, and light reactions by decreased proteins (Figure 4F). Although the changing trend of DAGs was slightly different from those in the WT, the regulatory network in *cgl1* was still detectable (Figure 4F). We also found several DAGs involved in oxidative stress response, including PRX32, PRX34, GGAT1, and RHS19 (Figures 4E,F), indicating that the peroxidase members responsible for cellular hydrogen peroxide clearance orchestrated with others to withstand salt stress.

### Biological Validation of Quantitative Proteomics Results

**Figure 5:**
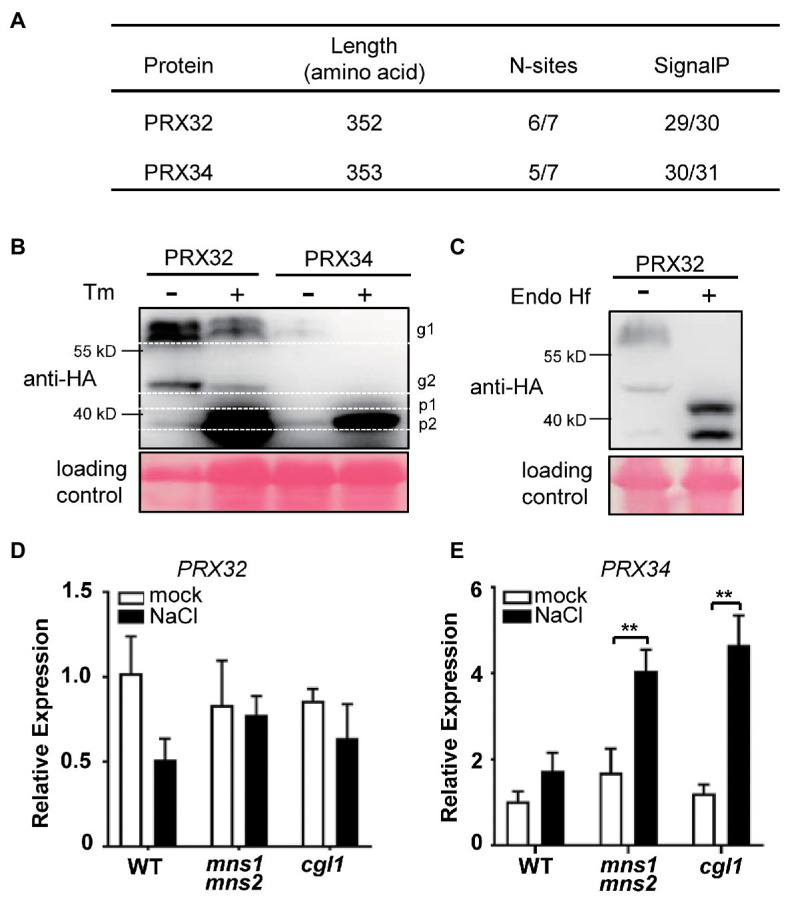
PRX32 and PRX34 are N-glycosylated, and their expressions are responsive to salt stress. **(A)** Prediction of N-glycosylation sites and signal peptides in PRX32 and PRX34. **(B)** N-glycosylation analysis of PRX32 and PRX34 proteins by tunicamycin treatment. PRX32-HA and PRX34-HA were transiently expressed in tobacco leaves in the absence or presence of tunicamycin and analyzed by immunoblots using anti-HA antibodies. g1 and g2 indicated two glycosylated bands; p1 and p2 represented two non-glycosylated bands. Ponceau S staining served as a loading control. The molecular weights of PRX32 and PRX34 are both 39 kD. **(C)** N-glycan analysis of PRX32 by Endo H digestion. The recombinant protein PRX32-HA was transiently expressed in tobacco leaves. After digested with glycosidase Endo Hf, the total proteins were separated on SDS-PAGE and detected with anti-HA antibodies. Ponceau S staining served as a loading control. **(D,E)** Quantitative real-time PCR (qPCR) analysis of gene expression in response to salt stress. Total RNAs were extracted from 2-week-old seedlings treated with or without salt stress described in Figure 1. The expression levels of *PRX32* **(D)** and *PRX34* **(E)** were analyzed by qPCR. ^**^Indicates a significant difference at *p* < 0.001.

**Figure 6:**
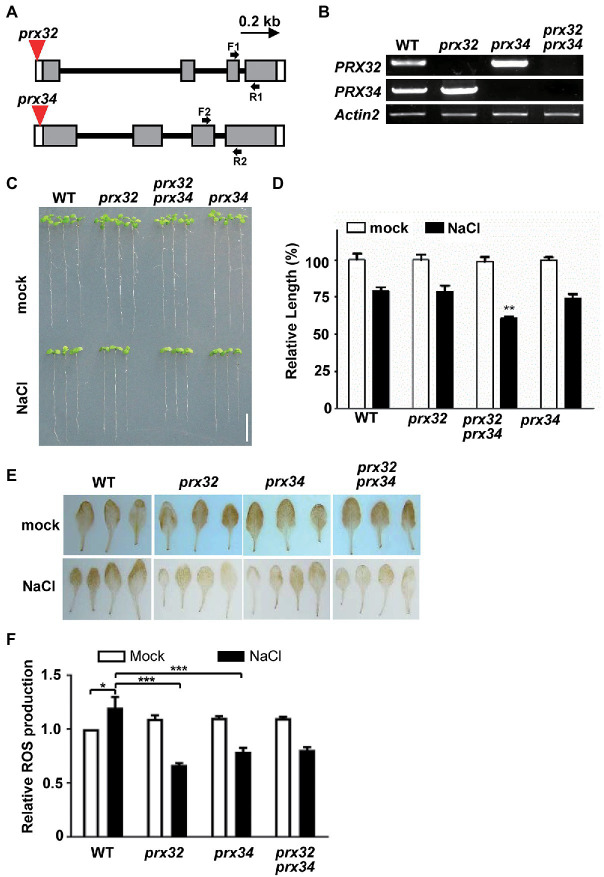
PRX32 and PRX34 are required for root growth under salt stress. **(A)** T-DNA insertion sites of *prx* alleles. Gray boxes represent exons and are linked with introns (lines). The open boxes denote untranslated regions (UTR). The location of the T-DNA insertion site in each mutant line is marked as a red triangle. A bar with an arrow from 5' to 3' representing 0.2 kb length is shown. **(B)** Reverse transcription-PCR (RT-PCR) analyses of *PRX32* and *PRX34* gene expression. Two-week-old *Arabidopsis* seedlings were used for RNA extraction, and the transcript of *Actin2* was amplified as an internal control. **(C)** Root growth phenotypes against salt treatment. Five-day-old seedlings were transferred to MS medium supplemented without (mock) or NaCl to grow for 9 days before taking photos. Bar = 0.5 cm. **(D)** Statistics analysis of root length. The root length shown in **(C)** was statistically analyzed. The primary root length of the WT (mock) was set as 100%, and relative values were shown. ^**^Indicated a significant difference from the value of the WT (NaCl) with *p* < 0.001, *n* > 20 (Student’s *t*-test). **(E)** Reactive oxygen species (ROS) production analysis by 3,3'-Diaminobenzidine (DAB) staining. Two-week-old seedlings were transferred to liquid Murashige and Skoog (MS) medium supplemented without (mock) or 200 mM NaCl to grow for 6 h before staining. **(F)** Statistics analysis of DAB staining. The measurement was performed in ImageJ software. The value of WT (mock) was set as 1.0, and relative values were shown. * and *** indicated significant differences at *p* < 0.01 and *p* < 0.0001, respectively (both *n* > 10, Student’s *t*-test).

To verify if the DAGs identified by the MS reflect the physiological responses of plants to salt stress, we select the candidate proteins for further validation. The GO functional analysis indicated that peroxidase directly or indirectly participated in the salt response (Table 2; Figure 4). The protein abundance of two peroxidases, PRX32 and PRX34, significantly increased in the salt stress-sensitive *cgl1* mutant (Table 2; Supplementary Table S5). These two proteins are predicted harboring signal peptides and more than five potential N-sites with the NetNGlyc server (Figure 5A).^6^ We transiently expressed PRX32 and PRX34 in tobacco leaves in the absence or presence of tunicamycin, an N-glycosylation inhibitor. As shown in Figure 5B, the obvious accumulations of under-glycosylated forms of PRX32 and PRX34 were observed from recombinant PRX32-HA and PRX34-HA proteins. Similarly, after digestion with glycosidase Endo H, the band shifts and the accumulation of deglycosylated PRX32-HA proteins were detected (Figure 5C), implying that both proteins were N-glycosylated *in vivo* and their abundances were regulated by associated N-glycan. To further test if *PRX32* and *PRX34* were salt-responsive genes, qPCR analyses were performed. The basal expression level of *PRX32* was low, and no obvious response to salt stress in WT and mutants was detected. By comparison, *PRX34* was significantly upregulated against salt stress in *mns1 mns2* and *cgl1* mutants (Figures 5D,E). We also used *Arabidopsis* T-DNA insertional lines to test the response of *prx32* and *prx34* to high salinity. The T-DNA insertion of both lines laid in the 5'-untranslated region (UTR; Figure 6A), and semi-quantitative RT-PCR confirmed no detectable transcripts from *prx32* and *prx34* mutants either (Figure 6B). The single mutant of *prx32* and *prx34* displayed similar primary root length as WT under salt stress. However, the double mutations of *prx32 prx34* were obviously sensitive to salt treatment, showing a further reduction in root length compared to the WT (*p* = 0.0009, *n* > 20; Figures 6C,D), suggesting that these two proteins might have overlapping effects on root growth under salt stress. In addition, DAB staining was conducted to explore whether the activity of PRX32 and PRX34 was relevant to the salt stress response. Agreed with a previous study that PRX34 functions in an apoplastic oxidative burst (O’Brien et al., 2012), H_2_O_2_ production induction by salt stress decreased *prx34*, *prx32*, and *prx32 prx34* double mutants as well (Figures 6E,F). These results suggested the crucial roles of two functionally redundant PRX proteins in the salt response. PRX32 and PRX34 belong to the class III peroxidase (PRX) multigene superfamily, containing 73 encoding genes in *Arabidopsis* (Valerio et al., 2004); however, their biological functions remain largely unknown. It is possible that more PRX proteins were involved in the salt response. In addition, the results suggested that the identified DAGs are potential biologically functional salt-responsive glycoproteins.

## Discussion

In the current study, we conducted an MS-based label-free proteomic analysis to study differentially abundant glycoproteins in *mns1 mns2* and *cgl1-3* mutants using lectin enrichment methods (Zielinska et al., 2010). The results showed that N-glycosylation biosynthesis was important for the homeostasis of the plant cell against high salinity. By analyzing DAGs, we found that the abundance of several subsets of glycoproteins responsible for glycoprotein biosynthesis, stress response, signal transduction, and oxidation-reduction process increased, while energy production and cell wall organization related glycoproteins were reduced under salt stress in the WT (Table 2; Figure 4F). In agreement with previous studies, our data suggested that plants primarily need to save energy for survival due to impaired photosynthesis system under stress conditions. However, the abundance modulation of salt-responsive glycoproteins was impaired in both mutants, presumably due to the lack of mature N-glycan. In addition, comprehensive studies between *mns1 mns2* and *cgl1* in response to high salinity revealed that two N-glycan modification steps mediated by MNS1/MNS2 and CGL1 might differentially modulate client glycoproteins, which is consistent with the difference of salt-sensitive phenotypes between *mns1mns2* and *cgl1-3* mutant (Liu et al., 2018). Thus, our results supply an integral overview of the requirements of highly coordinated N-glycosylation steps in plants to adapt to environmental stress.

Reactive oxygen species (ROS), including the superoxide anion, hydrogen peroxide, and hydroxyl radical, are normal byproducts of aerobic cell metabolism, but a large amount of ROS production and accumulation induced by salt stress in plant cells is dangerous for photosynthesis, cell wall biosynthesis, and many other biological processes (Munns and Tester, 2008; Yang and Guo, 2018). The active oxygen scavengers in plants include catalase (CAT), ascorbic acid peroxidase (APX), superoxide dismutase (SOD), glutathione reductase (GR), and some non-enzymatic molecules such as anti-ascorbic acid, glutathione, carotenoids, and anthocyanins (Apel and Hirt, 2004). *Arabidopsis* contains a complex ROS synthesis, regulation, and clearance-related network involving more than 150 genes (Mittler et al., 2004). Our results demonstrated that two functional peroxidases, PRX32 and PRX34, were potentially modulated by N-glycosylation and responsible for root development and salt sensitivity (Figures 5, 6). However, the exact roles of PRX32 and PRX34, together with other class III peroxidase members in maintaining root development against salt stress, still need to be investigated (Arnaud et al., 2017). Moreover, addressing the regulatory roles of their attached N-glycans is another challenge.

In the present study, we demonstrated that the protein abundance changes in *mns1 mns2* and *cgl1-3* differed by label-free quantitative analysis (Table 2). GO and KEGG pathway results also indicated that the modulated substrate proteins in *mns1 mns2* and *cgl1-3* were involved in different biological pathways under salt stress (Figure 3; Supplementary Figure S5). We suspected that each N-glycosylation step might influence the specific subsets of glycoproteins during stress responses. Several reports have shown that different N-glycoproteins may harbor different N-glycan structures. For instance, TGG1, one of two myrosinase enzymes in *Arabidopsis*, is decorated with oligomannosidic N-glycans, as seen in the MS (Liebminger et al., 2012). However, most attached N-glycans on the RSW2 protein, which is critical for cell wall synthesis, were modified with a complex glycan structure (Liebminger et al., 2013). The difference in N-glycan structures between TGG1 and RSW2 may reflect intrinsic protein structures for native cellular functions and may be the molecular basis for differential regulation by N-glycosylation steps.

Distinct from the case of responses to biotic stress, plant responses to abiotic stress often involve multiple genes in multiple metabolic pathways, and there is a complex association between different abiotic stress responses (Cabello et al., 2014). From the perspective of genomics and proteomics, the regulatory network of abiotic stress responses exhibits temporal and spatial complexity in the context of signal transduction and expression of regulatory genes and functional proteins such as transcription factors and enzymes responsible for stress-related metabolite synthesis or deposition (Zhu, 2016). In addition, the protein-quantification method using MS coupling label-free analysis may lead to different protein quantification results due to the differing abundance of N-glycopeptides identified from the same protein (Song et al., 2013). Therefore, it will be helpful to integrate valuable findings from different large-scale multi-omics experiments. Recently, several studies revealed the N-glycan microheterogeneity on glycoproteins in *Arabidopsis* (Xu et al., 2016; Zeng et al., 2017). It would be interesting to understand whether N-glycosyl regulation occurs under specific conditions. It is necessary to determine the N-glycan structures and quantitation on the glycopeptides to evaluate the precise roles of each N-glycan in the future.

## Data Availability Statement

The datasets presented in this study can be found in online repositories. The names of the repository/repositories and accession number(s) can be found at: http://www.proteomexchange.org/, PXD006893.

## Author Contributions

CL and ZH conceived and designed the experiments. CL, HZ, HC, DH, and XL performed the experiments. CL, GN, and PL conducted the bioinformatic analysis. CL and GN wrote the manuscript. PL and ZH revised the manuscript. All authors contributed to the article and approved the submitted version.

### Conflict of Interest

The authors declare that the research was conducted in the absence of any commercial or financial relationships that could be construed as a potential conflict of interest.

## Footnotes

^1^http://bioinfo.cau.edu.cn/agriGO/

^2^http://www.genome.jp/kegg/

^3^http://string-db.org/

^4^https://imagej.nih.gov/ij/download.html

^5^https://string-db.org

^6^http://www.cbs.dtu.dk/services/NetNGlyc/
