# Supplementary material for: Comparative Label-Free Quantitative Proteomics Analysis Reveals the Essential Roles of N-Glycans in Salt Tolerance by Modulating Protein Abundance in Arabidopsis
Source: Front Plant Sci. 2021 Jul 2;12:646425. doi: 10.3389/fpls.2021.646425 (PMC8283305; doi:10.3389/fpls.2021.646425)
Supplement: Supplementary file 5 [file Data_Sheet_1.PDF]

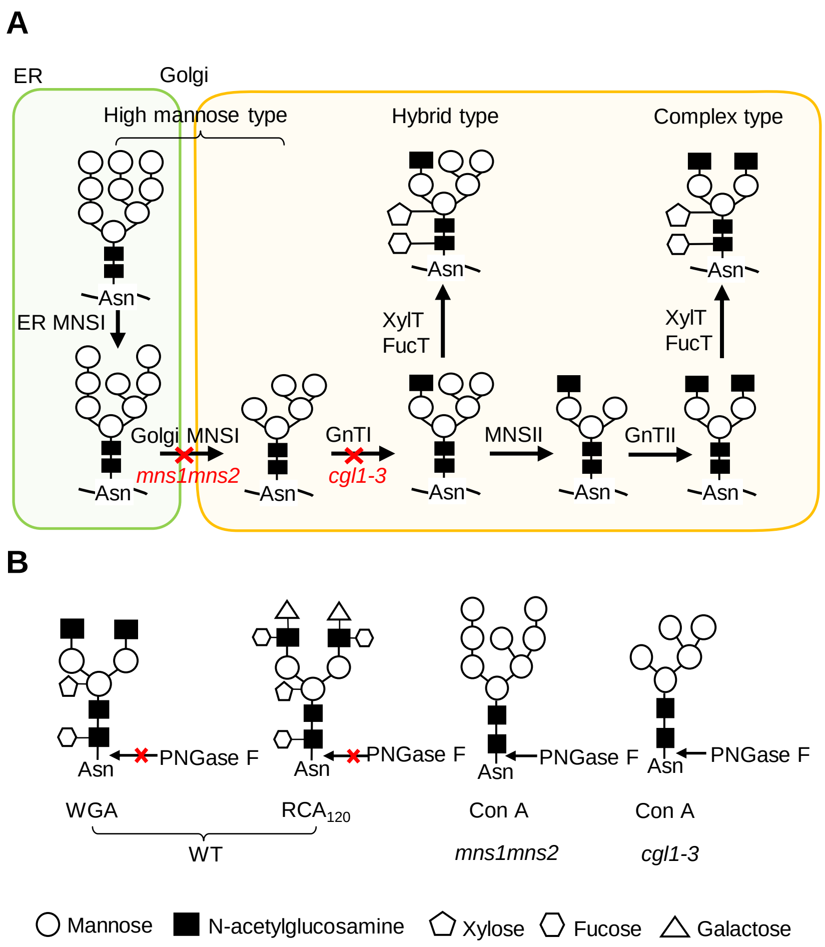


**Figure S1. Proposed dominant N-glycan structures in WT and mutant plants.**
**(A)** Proposed N-glycosylation pathway in plants. N-glycan precursor is transferred to nascent proteins by OST complex in ER. Two Golgi MNSI enzymes trim three α1,2-Man residues from the N-glycan prior to CGL1 catalyzed add of a GlcNAc. In Golgi, hybrid and complex type N-glycan are derived from high mannose type N-glycan. The mutant lines used in this study and their defective steps are shown in red font. **(B)** Analysis of mature and defective oligomannosidic N-glycans in WT, *mns1 mns2* and *cgl1-3*, respectively. Glycopeptides are enriched by attached N-glycans via recognizing by different type of lectins. N-glycans containing α1,3-fucose are hardly digested from glycopeptides collected from WT samples by PNGase F.

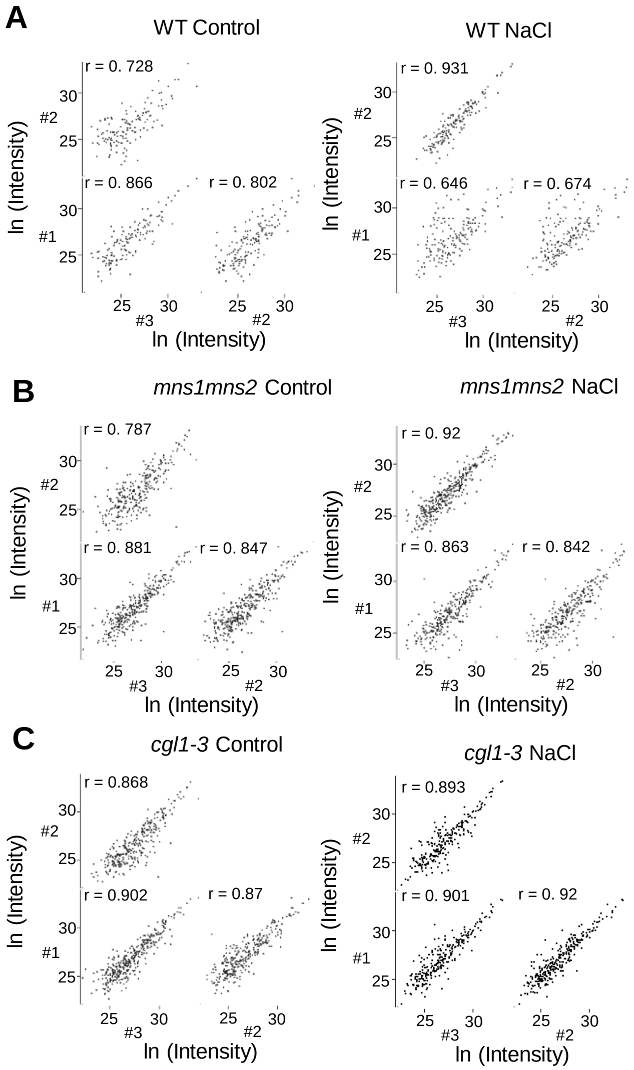


**Figure S2. Quality evaluation of label-free MS quantitation results from three repetitions.**

Pearson correlation analysis of N-Glycopeptide Intensity in Control or NaCl treated samples of WT **(A)**, *mns1 mns2* double mutant **(B)**, and *cgl1-3* **(C)** in three biological replicates, respectively. The natural logarithm of N-glycopeptide Intensity is used for analysis, and the correlation coefficient *r* within two biological replicates is shown.

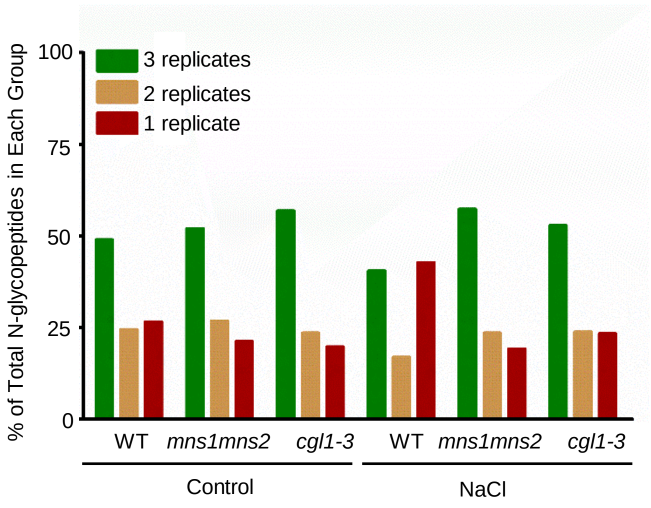


**Figure S3. Quality assessment of the N-glycoproteomics results by N-glycopeptides number.** The percentages of N-glycopeptides identified in three, two, or one of the replicate analyses are shown for three *Arabidopsis* lines in control and NaCl groups, respectively.

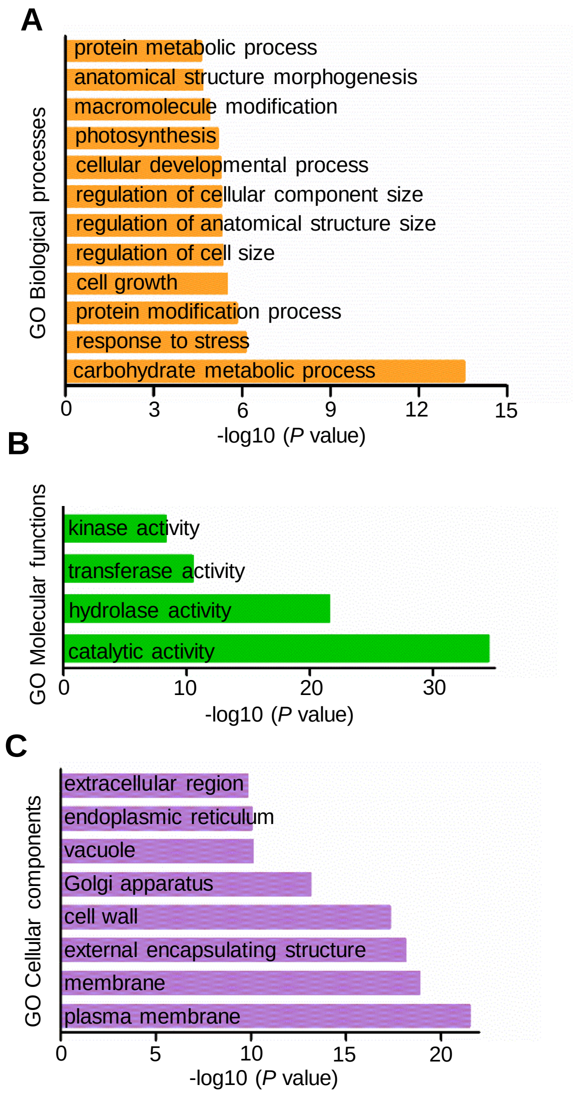


**Figure S4. Gene Ontology analysis of total glycoproteins identified by LC-MS/MS.** Colorful boxes indicate items from biological processes (orange, **A**), molecular functions (green, **B**) and cellular components (purple, **C**) that are significantly overrepresented in the N-glycoproteome compared to the entire *Arabidopsis* proteome according to Gene Ontology analysis at website http://bioinfo.cau.edu.cn/agriGO/analysis.php, respectively.

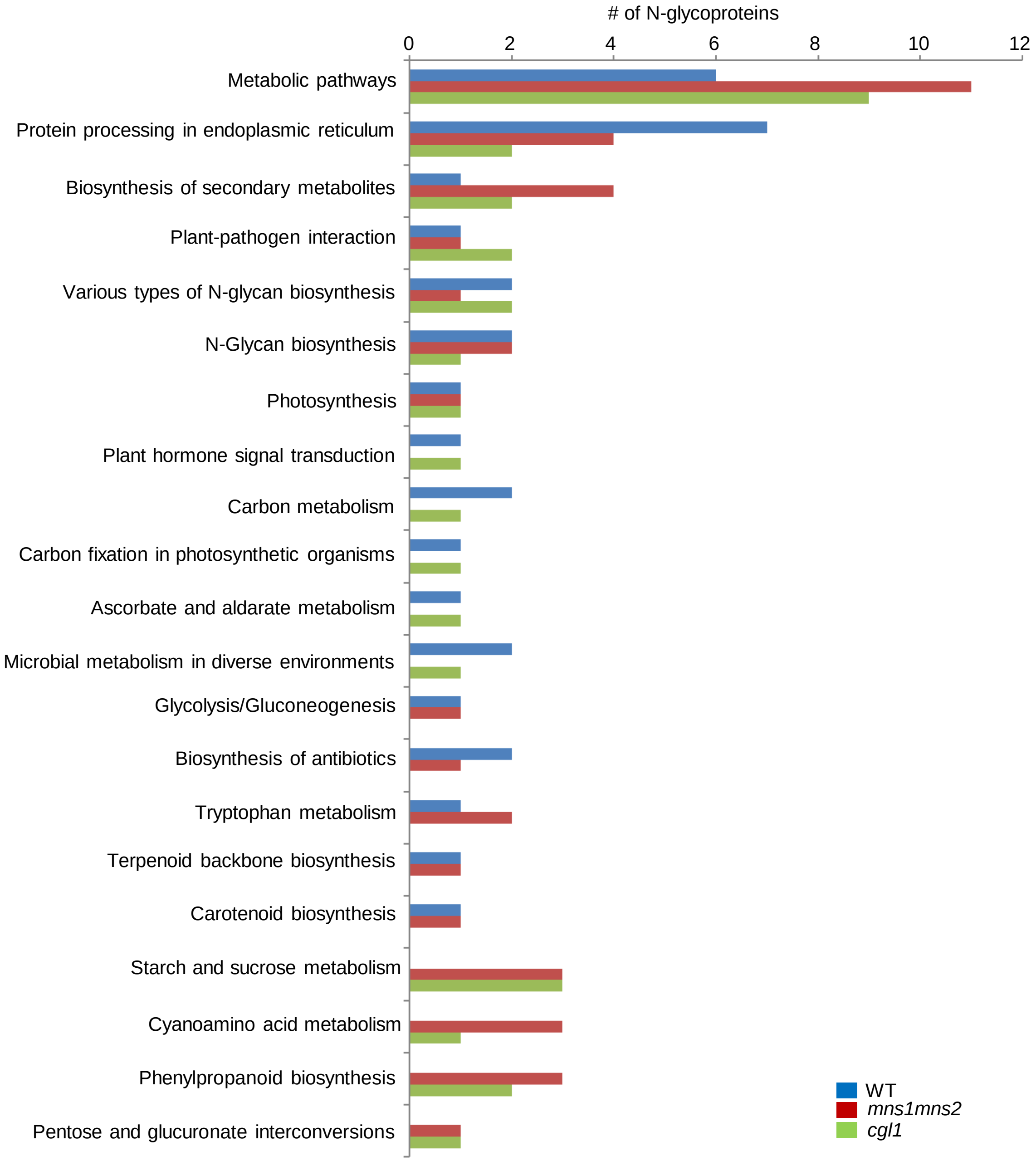


**Figure S5. Different KEGG pathways were modulated in WT and N-glycan maturation mutants.** Salt responsive glycoproteins identified from WT, *mns1 mns2* and *cgl1-3* were analyzed by KEGG website server at http://www.genome.jp/kaas-bin/kaas_main, respectively.
